# Deep learning framework for automated goblet cell density analysis in in-vivo rabbit conjunctiva

**DOI:** 10.1038/s41598-023-49275-y

**Published:** 2023-12-21

**Authors:** Seunghyun Jang, Seonghan Kim, Jungbin Lee, Wan Jae Choi, Chang Ho Yoon, Sejung Yang, Ki Hean Kim

**Affiliations:** 1 Department of Biomedical Engineering, Yonsei University, 1 Yonseidae-gil, Wonju-si, Gangwon-do, 26493 Republic of Korea; 2 Department of Precision Medicine, Yonsei University Wonju College of Medicine, 20 Ilsan-ro, Wonju-si, Gangwon-do, 26426 Republic of Korea; 3 Department of Medical Informatics and Biostatistics, Graduate School, Yonsei University, 50-1 Yonsei-ro, Seodaemun-gu, Seoul, 03722 Republic of Korea; 4 Department of Mechanical Engineering, Pohang University of Science and Technology, 77 Cheongam-ro, Nam-gu, Pohang, Gyeoungbuk 37673 Republic of Korea; 5 Department of Ophthalmology, Seoul National University College of Medicine, 101 Daehak-ro, Jongno-gu, Seoul, 03080 Republic of Korea; 6 Laboratory of Ocular Regenerative Medicine and Immunology, Biomedical Research Institute, Seoul National University Hospital, 101 Daehak-ro, Jongno-gu, Seoul, 03080 Republic of Korea

**Keywords:** Cellular imaging, Image processing, Eye manifestations

## Abstract

Goblet cells (GCs) in the conjunctiva are specialized epithelial cells secreting mucins for the mucus layer of protective tear film and playing immune tolerance functions for ocular surface health. Because GC loss is observed in various ocular surface diseases, GC examination is important for precision diagnosis. Moxifloxacin-based fluorescence microscopy (MBFM) was recently developed for non-invasive high-contrast GC visualization. MBFM showed promise for GC examination by high-speed large-area imaging and a robust analysis method is needed to provide GC information. In this study, we developed a deep learning framework for GC image analysis, named dual-channel attention U-Net (DCAU-Net). Dual-channel convolution was used both to extract the overall image texture and to acquire the GC morphological characteristics. A global channel attention module was adopted by combining attention algorithms and channel-wise pooling. DCAU-Net showed 93.1% GC segmentation accuracy and 94.3% GC density estimation accuracy. Further application to both normal and ocular surface damage rabbit models revealed the spatial variations of both GC density and size in normal rabbits and the decreases of both GC density and size in damage rabbit models during recovery after acute damage. The GC analysis results were consistent with histology. Together with the non-invasive high-contrast imaging method, DCAU-Net would provide GC information for the diagnosis of ocular surface diseases.

## Introduction

Goblet cells (GCs) in the conjunctiva are specialized epithelial cells that secrete mucins on the ocular surface. Mucins from GCs form the innermost mucus layer of tear film, and the mucus layer spreads the tear film for protection. GC dysfunction or loss is associated with various ocular surface diseases including dry eye disease^1,2^. Therefore, GC examination is important for both diagnosis and treatment monitoring. However, GC examination has been difficult due to the limitations of current examination methods. Impression cytology (IC) and reflection confocal microscopy (RCM) have been used for GC examination in human subjects. IC is a simple examination method that removes superficial cells including GCs with filter papers. GCs on the filter paper are visualized after various histological processes including periodic acid-Schiff (PAS) staining. IC has several limitations of long examination time, lack of standardization, and invasiveness, etc. RCM is a non-invasive 3D imaging method based on light reflection. RCM was used to visualize various cells in the eye, and an RCM study of conjunctival CGs was previously conducted^3^. However, RCM has limitations of low image contrasts, small fields of view, and inconvenient contact imaging. We recently developed a high-contrast GC imaging method, called as moxifloxacin-based fluorescence microscopy (MBFM)^4–6^. MBFM is a fluorescence imaging method based on specific GC labeling of a Food and Drug Administration (FDA)-approved moxifloxacin ophthalmic solution. MBFM demonstrated high-contrast CG visualization in various live animal models and the imaging was non-invasive by using excitation energy much less than the phototoxicity threshold. MBFM can be applied to human subjects for GC examination. A robust image analysis method needs to be developed to make GC information available for diagnosis.

There are various cell image analysis methods. Conventional methods use basic image processing algorithms for cell segmentation such as intensity thresholding, filtering, morphological operations, and deformable model fittings^7–9^. A region growing method expanding seed points for segmentation was successfully used in some studies^10,11^. However, conventional methods often faced challenges, requiring continuous parameter adjustments dealing with new dataset, making it laborious to achieve consistent results. With the advent of deep learning methods, particularly those using convolutional neural networks (CNNs), these shortcomings have been addressed^12,13^. CNNs demonstrated their effectiveness in automating biomedical image analysis by learning the characteristics. Deep-learning methods include regression- and detection-based methods that learn the number and morphology of cells, respectively^14–16^. Detection-based methods are useful for detail cell analysis^17^. U-Net is a widely used detection-based method in medical image analysis by exhibiting high pixel-level discrimination and good segmentation performance on small data sets^18^. Enhanced U-Net models introduced novel attention methods to skip connections for selective integration of encoder information with decoders^19–22^. Because GCs are densely distributed either individually or in clusters on the conjunctival surface with irregular background shadowed by underlying blood vessels, it is necessary to develop a feature extraction method optimized for the characteristics of GC images.

In this study, we propose a dual-channel attention U-Net (DCAU-Net) that employs dual-channel convolution and a novel global channel attention mechanism for robust GC segmentation from MBFM GC images. There are two main contributions. First, we developed DCAU-Net which had a modified encoder to extract features with dual-channel modules and a modified skip connection to retain the global information. The dual-channel modules integrated semantic and texture feature information and delivered it to the next layer. The global channel attention module strengthened feature representation and reduced redundancy. Second, we applied DCAU-Net to the GC analysis in MBFM images of live rabbit models in comparison with PAS labeled histological images in terms of both GC density and morphological characteristics. GC density from DCAU-Net was compared with that from the manual counting by experts in MBFM images. Moreover, DCAU-Net was applied to GC analysis in MBFM images of ocular surface damage rabbit models to visualize their changes.

## Results

### Performance comparison between DCAU-Net and U-Net in GC segmentation

**Figure 1:**
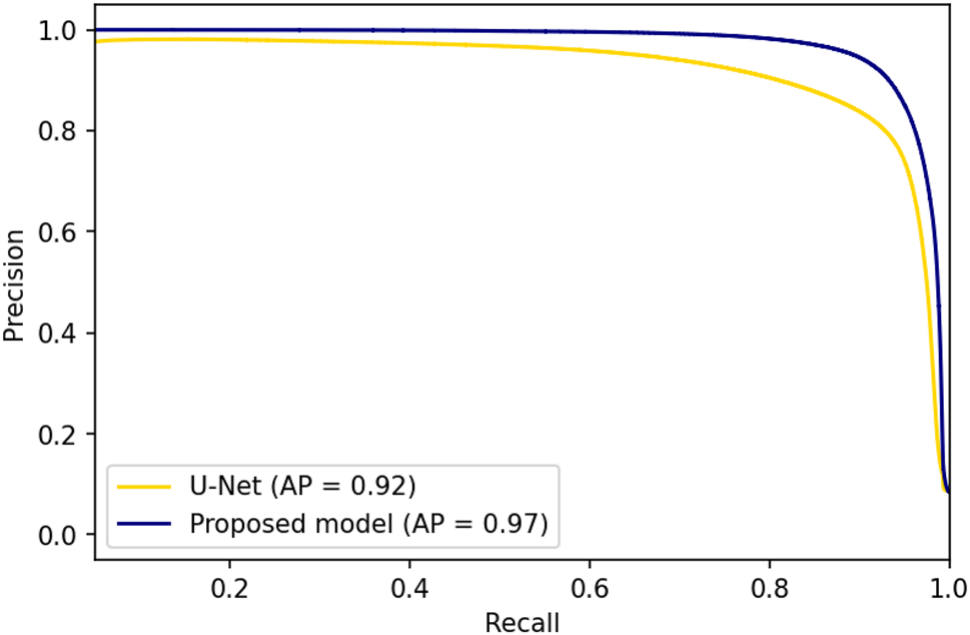
Comparison of the precision-recall curves of the standard U-Net and DCAU-Net models. *AP* the area under precision-recall curve.

The performance of DCAU-Net in GC segmentation was evaluated in comparison with the standard U-Net model. The evaluation results are summarized in Table 1 and Fig. 1.

**Table 1:** Dice, intersection over union (IoU), recall and precision scores of U-Net and DCAU-Net in the segmentation of GCs in MBFM images of normal rabbit models.

Metrics	U-Net	DCAU-Net
Dice coefficient	0.896 ± 0.149	0.931 ± 0.004
Intersection over union (IoU)	0.825 ± 0.230	0.871 ± 0.008
Recall	0.947 ± 0.034	0.931 ± 0.016
Precision	0.858 ± 0.034	0.932 ± 0.017

The comparison testing was repeated 5 times, and DCAU-Net achieved better performance in dice coefficient and IoU than the standard U-Net. The standard U-Net exhibited imbalanced results with relatively high recall and low precision scores compared to DCAU-Net. The precision-recall curve was used to indicate the discriminating ability of these models with respect to the variation in discrimination threshold. It was an evaluation index that did not contain true negatives. For recall values of 0.8 or higher, DCAU-Net showed higher precision values than U-Net.

**Figure 2:**
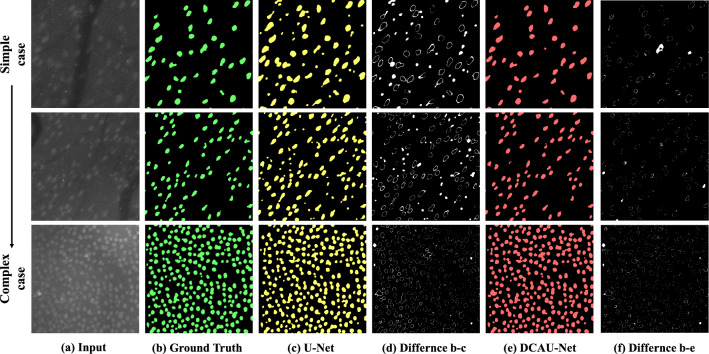
Comparison of original U-Net and DCAU-Net in the segmentation of GCs at different densities. (**a**) Raw image. (**b**) Image GT. (**c**) U-Net. (**d**) Difference between GT and U-Net. (**e**) DCAU-Net. (**f**) Difference between GT and DCAU-Net.

The segmentation results of U-Net and DCAU-Net in three representative GC images with different cell densities are presented in Fig. 2. Input GC images, GT images, U-Net segmentation images and their difference images with GT images, DCAU-Net images and their difference images are presented in different columns. Both U-Net and DCAU-Net segmented GCs well in all three GC images in general. The difference images revealed that U-Net tend to segment GCs smaller than GT images. U-Net also missed some GCs and misclassified non-GC objects with strong signals as GCs. U-Net did not resolve aggregated GCs, however, DCAU-Net segmented GCs better and had lower rates of missing and misclassification than U-Net. Therefore, DCAU-Net performed better than the standard U-Net in GC segmentation.

### GC density analysis with DCAU-Net in normal rabbit models

**Figure 3:**
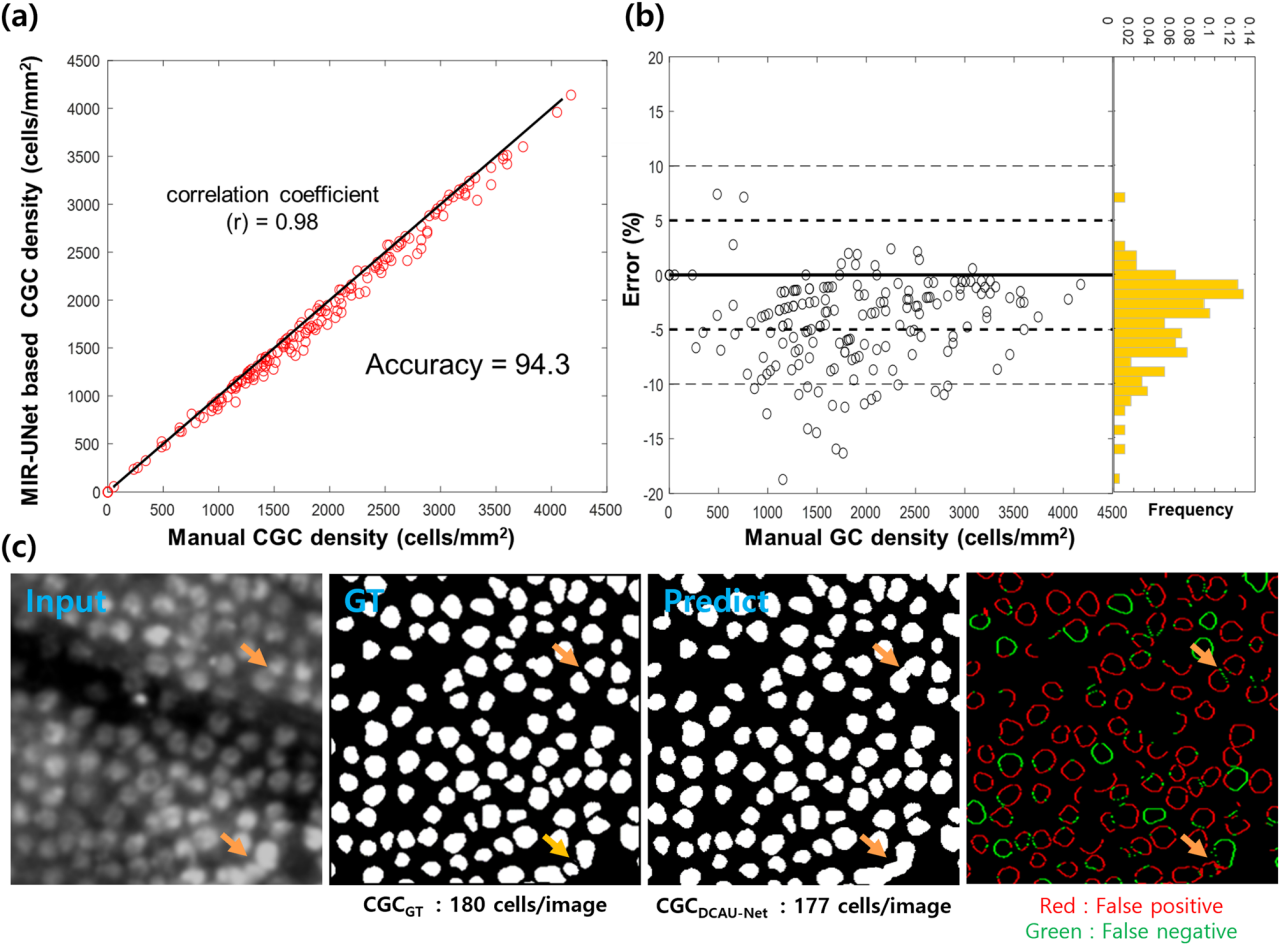
DCAU-Net application to GC density analysis in MBFM images of live normal rabbit models in comparison with manual counting by experts. (**a**) A scatter plot presenting GC densities obtained from DCAU-Net and the ones from expert manual counting. (**b**) A scatter plot presenting GC density errors which were obtained via (GC densities from DCAU-Net) − (GC densities from expert counting). (**c**) A representative MBFM input image, GT image, DCAU-Net prediction image, and difference between GT and prediction images. Orange arrows in (**c**) mark aggregated GCs.

DCAU-Net was applied to GC density analysis of normal rabbit models. GC densities were obtained by counting the number of segmented GCs per unit area. GC densities from DCAU-Net segmentation were compared with GC densities from manual counting by plotting the pair densities in a 2D scatter plot as shown in Fig. 3a. GC densities in various conjunctival regions including the fornix and bulbar conjunctiva were analyzed. The obtained GC densities varied in a large range from 1050 ± 640 to 2350 ± 630 cells/mm^2^, depending on the conjunctiva location. GC densities from DCAU-Net showed a linear relationship with those from manual counting with a correlation coefficient value of 0.98. As illustrated in Fig. 3b, the estimated GC densities were 94.3% of the true GC densities on average, and 95% of all GC density estimations were within − 12.4% and + 2.0% of the true GC densities. The slight underestimation of GC density with DCAU-Net was due to the counting of aggregated GCs as ones, as depicted in the representative images in Fig. 3c.

### Spatial variation of GC density and size in normal rabbit conjunctiva

**Figure 4:**
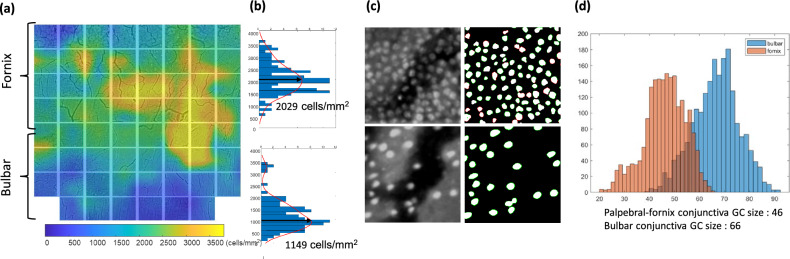
Analysis of GC density and size variations in the upper conjunctiva of a normal rabbit model, ex-vivo. DCAU-Net was used in the GC analysis. (**a–c**) Histograms of GC densities in the fornix and bulbar conjunctiva, a map of GC densities in the upper conjunctiva together with representative input and predicted GC images in the fornix and bulbar conjunctiva. (**d**) A histogram of GC sizes in the fornix and bulbar conjunctiva.

DCAU-Net segmentation helped to accurately estimate the GC density in MBFM images of live rabbit models. Automated GC density analysis was applied to mosaic MBFM images to visualize the spatial variation of GC density in the entire upper conjunctiva of ex-vivo normal rabbit models. A representative mosaic MBFM image covering a 20 × 20 mm^2^ conjunctival region is presented in Fig. 4a, and a GC density map in a different colormap was overlaid on the mosaic MBFM image. The mosaic GC image was generated by combining equally spaced 8 × 8 MBFM images. The GC density map indicated high spatial variations of GC density depending on the location in the upper rabbit conjunctiva. It was the highest (2030 ± 620 cells/mm^2^) in the fornix conjunctiva and the lowest (1150 ± 630 cells/mm^2^) in the bulbar conjunctiva (Fig. 4b). In the fornix region, GC density was higher on the nasal side than on the temporal side. These results were consistent with those of the previous studies^23,24^. DCAU-Net segmentation was used to analyze the size of GCs in addition to their density. The analysis results revealed a spatial variation of GC size depending on the conjunctival regions (Fig. 4c,d). GC sizes were 66 and 46 μm^2^ on average in the bulbar and fornix conjunctivas, respectively. GCs in the fornix conjunctiva were smaller and denser than those in the bulbar conjunctiva. The size variation of GCs has not been reported before and needs to be verified by statistical analysis of conventional PAS histological images in the future. The eccentricities of GCs in the bulbar and fornix conjunctiva were 0.54 and 0.49, respectively, indicating no significant difference.

### GC analysis in ocular surface damage rabbit models

**Figure 5:**
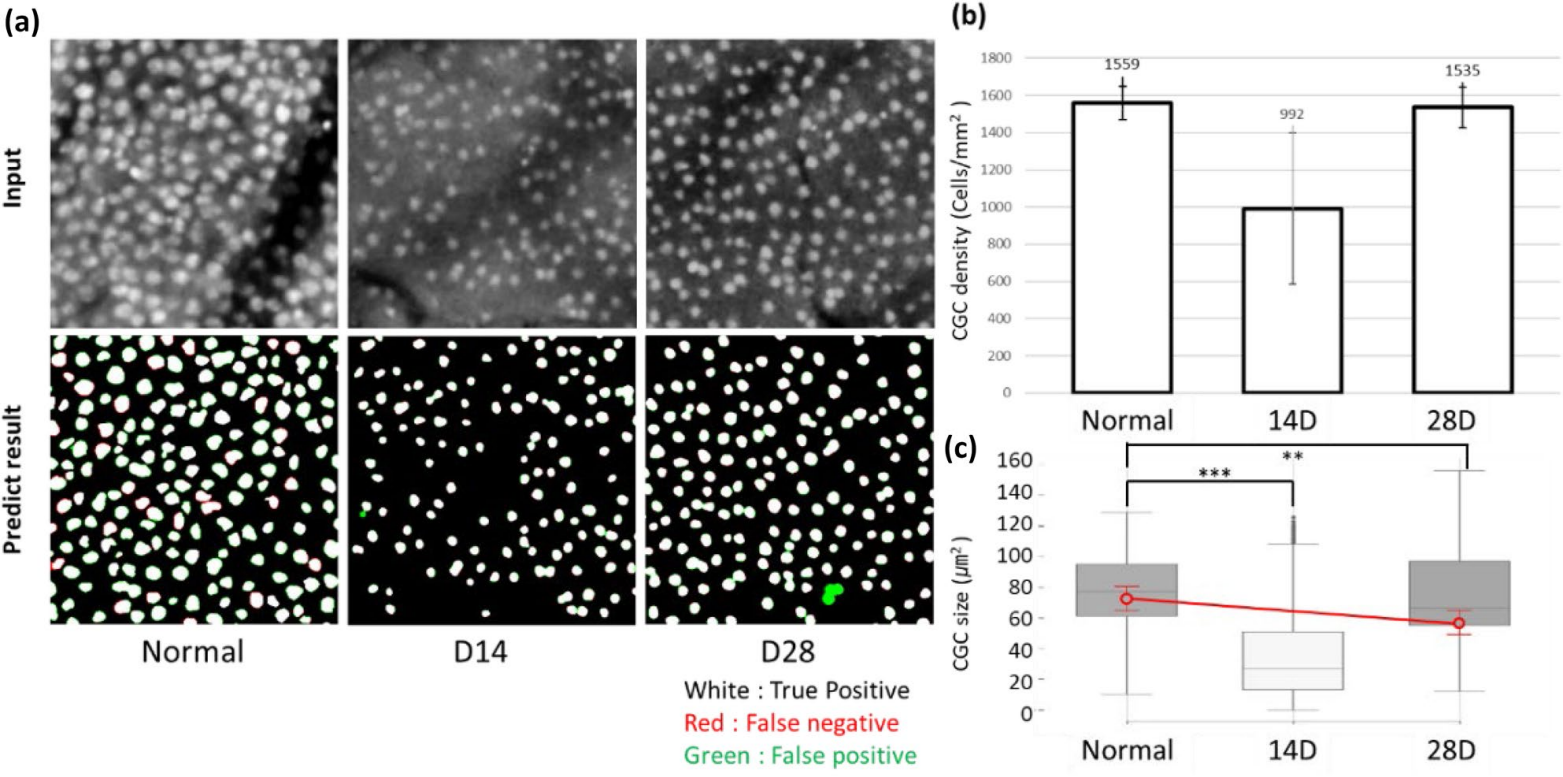
Longitudinal changes of GCs in ocular surface damage rabbit models during recovery from acute damage. (**a**) Representative MBFM images and DCAU-Net images at 3 different time points of before damage (normal), 14 and 28 days post damage. (**b**) GC densities at 3 time points. MBFM images in the bulbar conjunctiva of 5 rabbit models were analyzed. (**c**) GC size at 3 time points, red plot: size comparison in PAS histology result at normal and recovered rabbit for 4 weeks.

DCAU-Net, which was trained with the GC images of normal rabbit models, was applied to the analysis of GC images of ocular surface damage rabbit models. Damage was induced by the topical instillation of povidone iodine (PI). PI is a disinfectant commonly used in general ophthalmic surgeries including cataract surgery for the preoperative prevention of endophthalmitis, and it is known to induce damage to epithelial cells on the ocular surface^25^. Ocular surface damage was induced by one-time PI instillation. Significant GC damage was observed in the first week, after which GCs recovered gradually in later weeks. As shown in Fig. 5, MBFM images in 2 and 4 weeks after damage were used to observe GC changes in the recovery phase. GCs in the bulbar conjunctiva were analyzed mainly owing to the ease of in-vivo assessment. In 2 weeks, GCs were relatively sparse compared to the control, indicating recovery in progression. GC density was measured to be 1000 ± 500 cells/mm^2^, lower than the density before damage (1560 ± 90 cells/mm^2^). The size of GCs in 2 weeks after damage was approximately 20 ± 20 μm^2^, significantly smaller than the size of GCs before damage (78 ± 14 μm^2^). In 4 weeks, GC density was 1540 ± 90 cell/mm^2^, the same level as normal. However, the size of GCs was 60 ± 20 μm^2^, smaller than normal. The decrease of GC density in 2 weeks after damage and the complete recovery to normal level in 4 weeks were consistent with a previous study^4,25^. To determine the difference in GC size, PAS histology was performed in rabbit models both before damage and 4 weeks after damage. On PAS histology, the size of GCs was 76 ± 11 μm^2^ before damage and 61 ± 12 μm^2^ in 4 weeks after damage. Although the GC size from PAS histology could be affected by deformation during sample preparation including fixation and holding, the tendency of GCs being smaller than normal in 4 weeks after damage was consistent.

## Discussion

DCAU-Net was developed for automatic segmentation of GCs from MBFM images of live rabbit models. DCAU-Net was trained with GC images of normal rabbit models and used for the analysis of GC density and size in both normal and ocular surface damage rabbit models. DCAU-Net exhibited a superior performance in GC segmentation by modifying the U-Net structure for MBFM GC images. DCAU-Net segmented individually distributed GCs correctly and was not confused with small non-GC objects caused by overstaining. Although it was able to resolve some aggregated GCs, DCAU-Net performed well in general in MBFM GC images of rabbit models.

GC density estimation based on DCAU-Net segmentation revealed the high accuracy of approximately 94%. A slight underestimation was due to the miscounting of closely spaced GCs. DCAU-Net based GC analysis exhibited high spatial variations of GC densities depending on the conjunctival regions, consistent with the literature. The high spatial variations of GC density indicated that large area imaging is required for reliable GC density measurement. DCAU-Net based GC analysis revealed a statistically significant variation of GC size depending on the conjunctival regions: with fornix 58 ± 12 μm^2^ and bulbar 100 ± 18 μm^2^. The spatial variation of GC size was consistent with the one of PAS histology results. Although DCAU-Net was trained with GC images of normal rabbit models, it could be used for GC image analysis of ocular surface damage rabbit models and its GC density estimation in the damaged rabbit models showed comparable accuracy to that in the normal rabbit models. DCAU-Net based GC analysis showed decreases of both GC density and size in the damage rabbit models in the recovery phase from acute damage.

DCAU-Net analysis of the MBFM images took approximately 1 min per image. Although the processing time was quite long, the processing could be completed in time for the diagnosis which would occur in more than 10 min after the examination. Faster image processing would be desirable in the future. The current MBFM system had 1 fps imaging speed. Although it was enough for imaging anesthetized rabbit models, the higher imaging speed is required to image awake human subjects with minimal motion artifact and discomfort. We recently developed a high-speed extended depth-of-field (EDOF) microscopy running at 15 fps and demonstrated real-time mosaic imaging in rabbit models^26^. For fast and accurate analysis of GC images from the high-speed microscopy, further studies are needed to reduce computational resources by applying lightweight algorithms to the proposed model.

In conclusion, DCAU-Net was developed for the robust and automated segmentation of conjunctival GCs in MBFM images of live rabbit models. DCAU-Net based GC analysis showed approximately 94% accuracy with a slight underestimation in GC density and revealed the spatial variation of both GC density and size depending on the conjunctival regions in normal rabbits and the decreases of both parameters in ocular surface damage rabbit models. In combination with non-invasive MBFM, DCAU-Net based GC analysis might have potential for non-invasive GC examination and precision diagnosis of ocular surface diseases.

## Methods

### Overview of DCAU-Net architecture

**Figure 6:**
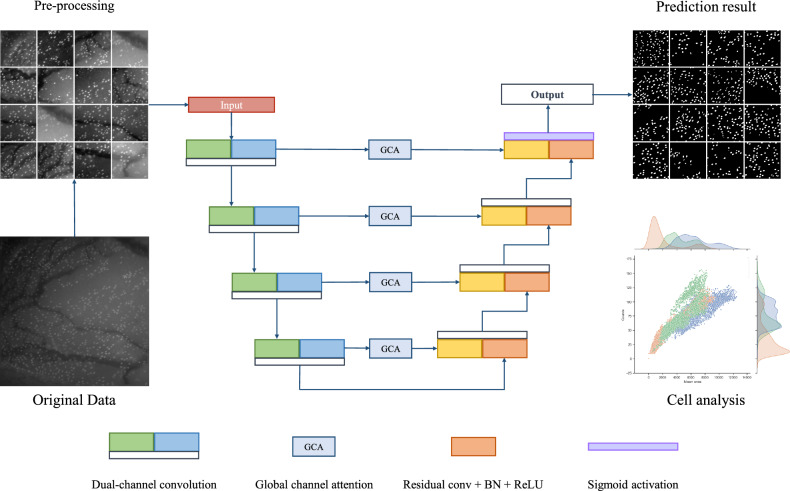
Overview of DCAU-Net. DCAU-Net is an end-to-end GC segmentation network that tracks GCs in the MBFM image. Each encoder consists of a dual-channel convolution (DCC) and executes propagation through the global channel attention (GCA).

The architecture of DCAU-Net is illustrated in Fig. 6. DCAU-Net had an encoder–decoder framework. The encoder consisted of dual-channel convolution (DCC) modules, which had a semantic channel and a texture channel. The semantic channel module was to extract features using a multi-scale kernel for small objects. The texture channel module had a large asymmetric filter to examine the overall texture information and cell boundaries. The global channel attention (GCA) module was introduced both to strengthen feature calibration and to propagate encoder information. GCA combined the spatial attention algorithm and channel-wise pooling.

### Dual-channel convolution (DCC) modules

**Figure 7:**
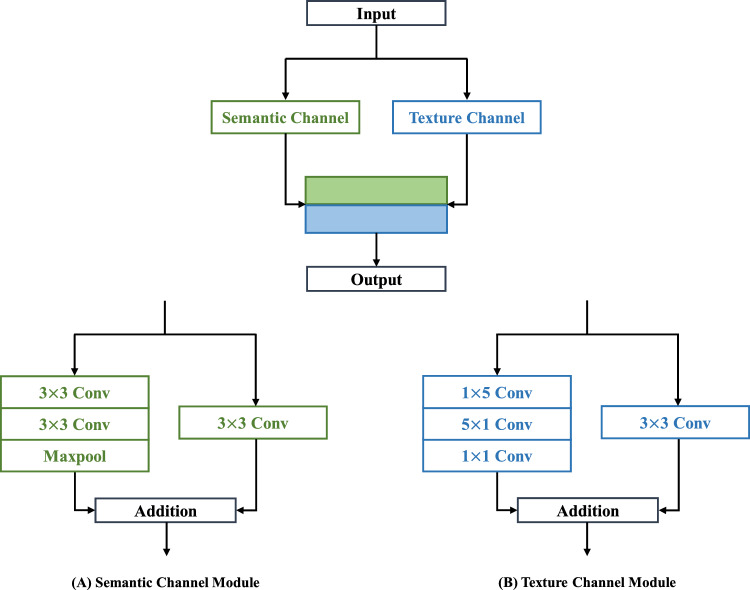
Overview of dual-channel convolution in the DCAU-Net. (**A**) Semantic channel module contains multi-scale convolution layers and a max-pooling layer. (**B**) Texture channel module utilizes asymmetric convolution kernels with stride controls.

Detailed DCC modules are illustrated in Fig. 7. The semantic channel module had multi-scale convolutions, as illustrated in Fig. 7a. The multi-scale convolution module had both a single 3 × 3 convolution layer and two 3 × 3 convolution layers in stack to obtain a wider receptive field than a single layer. Each convolution layer consisted of a stack of layer components, including residual convolution layers, batch normalization (BN) layers, and Rectified Linear Unit (ReLU) activation layers^27^. A max-pooling layer was added to the stacked layer to serve the purpose of downsampling the feature maps, reducing their spatial dimensions while retaining essential information, and extracting the maximum value from local regions to improve computational efficiency^28^. These layers maintained nonlinear properties to prevent internal covariate shift in the propagation process and to stabilize the gradient^29,30^. The texture channel module was used to obtain adaptive feature information. The texture channel module made dense predictions per pixel based on large kernel sizes^31^. The texture channel consisted of two convolution layers, as presented in Fig. 7b. The asymmetric convolution kernels were used to capture large receptive fields, enhancing the model to recognize textures and patterns with varying orientations while reducing the number of parameters.

### Global channel attention module (GCA)

**Figure 8:**
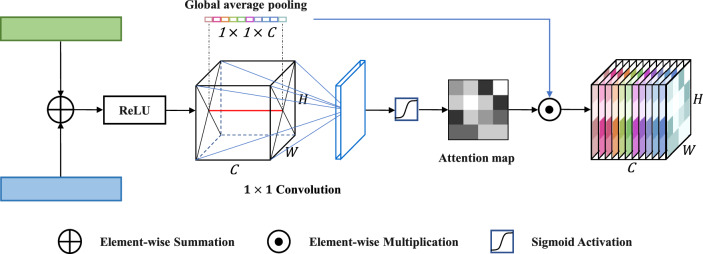
Structure of global channel attention module.

The skip connection of the DCAU-Net was modified with a global channel attention (GCA) module. The GCA module was designed to highlight features that propagate to the decoder path, as illustrated in Fig. 8. The semantic and texture features of DCC were added element-wise to fuse information of the extracted convolutional feature map. The GCA performed a squeeze operation to element-wise fused feature map ${I}{\prime}\in {R}^{C\times H\times W}$, where *C*, *H*, and *W* represent the dimensions of the ${I}{\prime}$ in terms of channels, height, and width, respectively. The module generated channel-wise descriptor ${I}^{s}\in {R}^{C\times 1\times 1}$ by employing global average pooling and aggregated the feature map ${I}{\prime}$ input to the GCA in the entire channel^32^.

1 $${I}^{s}=1/\left(H\times W\right){\sum }_{1}^{H}{\sum }_{1}^{W}{I}{\prime}\left(i,j\right).$$

An attention map ${I}^{A}\in {R}^{1\times H\times W}$ was modified through the sigmoid activation connected to the 1 × 1 convolution. Although ${I}^{A}$ condenses the channel depth information with the pointwise 1 × 1 convolution, it maintained the same size and height of the input data. Accordingly, these features were refined using matrix multiplication to form a channel-wise attention map. The final output of the module was obtained by

2 $${I}^{R}= \sigma \left({I}^{A}\right)\times {I}^{s},$$

where σ is the sigmoid activation. The refined information ${I}^{R}\in {R}^{C\times H\times W}$ was multiplied to the encoder features and propagated to the decoder features.

### Ground truth production process

In producing ground truth (GT), three steps were taken to minimize manual processes and to increase accuracy: (1) to produce the initial GT (cell and background binary image), (2) to generate the GT prediction using standard U-Net, (3) to produce the final GT with manual refinement.

*Step 1* Initial GT was created by manually setting thresholds on the image to distinguish bright GCs and background including blood vessels. Blood vessels underneath the conjunctival surface appeared in relatively low intensity levels due to both absorption and scattering of excitation light. Given the spatial variation in brightness of background and GCs, we divided the image into sections manually and adjusted the threshold adaptively to account for variations in goblet cell-background contrast^33^ (Supplementary Fig. 1A).

*Step 2* Initial GT was cropped to 512 $\times$ 512 pixels, augmented through rotation, flip, and random crop processes, and used for the training of standard U-Net (Supplementary Fig. 1B) For standard U-Net, the network parameters of the published paper were applied as is^18^. 6 rabbit data was used (6 original data) in the training. The standard U-Net was trained for 50 epochs and the Adam optimizer with a learning rate of 0.0001 was employed. The rough prediction results of 16 rabbit data were obtained using the trained standard U-Net.

*Step 3* Manual denoising processes, such as dividing clumped cells, annotating missing cells and removing incorrected identified cells, were employed to produce the final GT.

### Model training

The input dataset was prepared by applying Contrast Limited Adaptive Histogram Equalization (CLAHE) application^34^ and augmented through random cropping the original 2048 × 2048 pixel images to 512 $\times$ 512 pixels. These resulting images were further processed with rotation and flipping transformations, as part of our training process. We did not apply these transformations during the test phase, ensuring a clear differentiation between training and testing procedures. Using 16 rabbit data images, after excluding those with defocus or rabbit motion artifacts, a total of 4858 augmented images were generated. Considering the small number of rabbit cases in the data, we trained DCAU-Net by using 5 fold cross validation. Data from 16 rabbits were divided into 5 fold, ensuring that data acquired from the same case were allocated evenly to the 5 fold. It was repeated 5 times by using a different fold as the validation set and the remaining 4 fold as the training set, yielding a mean validation dice score of 0.913. We conducted evaluation by dividing the dataset into 4138 training images and 720 test images. This dataset division was utilized to retrain our model from scratch. To cope with the segmentation task, the loss function was constructed by combining dice and focal losses with weights of 0.7 and 0.3, respectively^35,36^.

### Experimental setup

#### Evaluation metric

Dice coefficient, sensitivity (SE), and precision were calculated to evaluate the performance of DCAU-Net. Additionally, the area under precision-recall curve was also employed. The pixels of the probability map were compared with the GT label, and classified into true positive (TP), false positive (FP), true negative (TN), and false negative (FN) in the confusion matrix.

3 $$False \, positive \, rate=1-\frac{TN}{TN+FP},$$

4 $$Recall=\frac{TP}{TP+FN}= True \, positive \, rate,$$

5 $$Precision=\frac{TP}{TP+FP},$$

6 $$Dice \, coefficient=2\times \frac{TP}{TP+FP+FN},$$

7 $$Intersection \, over \, union = \frac{TP}{TP+FP+FN}.$$

Regions of individual GCs were captured in the binarized probability map. Cell density and morphology studies were performed. In the morphology study, cell area and eccentricity were analyzed.

#### Training detail

For training, both the standard U-Net and DCAU-Net models were trained for 100 epochs each, and the Adam optimizer with a learning rate of 0.0001 was employed^37^. With a step decay scheduler adjusting the learning rate every 20 epochs, we also implemented early stopping. For the standard U-Net model, binary cross-entropy loss was employed, while for our proposed model, the combination of dice loss and focal loss was used as the loss function. Training was implemented on an Intel i9-10900 CPU @ 2.80 GHz desktop with NVIDIA 3090 graphics processor unit (GPU) using Pytorch version 1.7.1, Cuda 11.0, and Python 3.8.5^38^.

#### Animal model preparation

Twenty-four New Zealand white female rabbits weighing between 3.0 and 3.6 kg were used: 15 rabbits for in-vivo normal models, 5 rabbits for PI ocular damaged model, 4 rabbits for PAS histology. Imaging of rabbit was conducted under anesthesia via subcutaneous injection of Tiletamine-zolazepam (Zoletil®, Virbac, Carros, France; 0.2 cc/kg) and Xylazine (Rompun®, Bayer AG, Leverkusen, Germany; 0.2 cc/kg) mixture. For the ocular damaged group, 5 mL of 5% povidone-iodine (PI) solution was topically instilled onto the conjunctiva and excessive solution was allowed to run freely. After 3 min incubation, the conjunctiva was rinsed with 5 mL of balanced salt solution (BSS, Alcon). Moxifloxacin solution was instilled and the eyelids were closed for 1 min.

#### Periodic acid Schiff (PAS) staining and conjunctival GC evaluation

The conjunctiva tissue was placed on a slide with the epithelial surface up. The mounted tissue was left in air for approximately 1 min for tight adhesion to the slide. The mounted conjunctiva was excised and fixed in 10% neutral buffered formalin over night at 4 °C. The tissue was hydrated with 100% to 70% alcohol solutions, and the hydrated slide was oxidized in 0.5% periodic acid solution for 10 min and rinsed in water for 3 min three times. After that, the slide was placed in Schiff reagent for 20 min and rinsed in water for 10 min three times. They were then dehydrated in alcohol and finally covered with histological mounting medium. En-face PAS-positive GC images were evaluated under a light microscope.

#### Ethics approval

The study protocol was approved by the Institutional Animal Care and Use Committee of the Seoul National University Biomedical Research Institute (IACUC No. 20-0143-S1A1) and was conducted in accordance with ARRIVE guidelines. This study was performed in accordance with relevant guidelines and regulations.

### Supplementary Information

Supplementary Information.

## Data Availability

The datasets used and/or analyzed during the current study are available from the corresponding author upon reasonable request.
