# Supplementary material for: Deep learning framework for automated goblet cell density analysis in in-vivo rabbit conjunctiva
Source: Sci Rep. 2023 Dec 21;13:22839. doi: 10.1038/s41598-023-49275-y (PMC10739799; doi:10.1038/s41598-023-49275-y)
Supplement: Supplementary file 1 — Supplementary Information. [file 41598_2023_49275_MOESM1_ESM.docx]

**SUPPLEMENTARY INFORMATION**

**From Deep Learning Framework for Automated Goblet Cell Density Analysis in In-Vivo Rabbit Conjunctiva**

**Ground truth production process**

In producing ground truth (GT), three steps were taken to minimize manual processes and to increase accuracy: (1) to produce the initial GT (cell and background binary image), (2) to generate the GT prediction using standard U-Net, (3) to produce the final GT with manual refinement.

Step 1: Initial GT was created by manually setting thresholds on the image to distinguish bright GCs and background including blood vessels. Blood vessels underneath the conjunctival surface appeared in relatively low intensity levels due to both absorption and scattering of excitation light. Given the spatial variation in brightness of background and GCs, we divided the image into sections manually and adjusted the threshold adaptively to account for variations in goblet cell-background contrast. (Supplementary Figure 1. A)

Step 2: Initial GT was cropped to 512×512 pixels, augmented through rotation, flip, and random crop processes, and used for the training of standard U-Net. (Supplementary Figure 1. B) For standard U-Net, the network parameters of the published paper were applied as is. 6 rabbit data was used (6 original data) in the training. The standard U-Net was trained for 50 epochs and the Adam optimizer with a learning rate of 0.0001 was employed. The rough prediction results of 16 rabbit data were obtained using the trained standard U-Net.

Step 3: Manual denoising processes, such as dividing clumped cells, annotating missing cells and removing incorrected identified cells, were employed to produce the final GT.


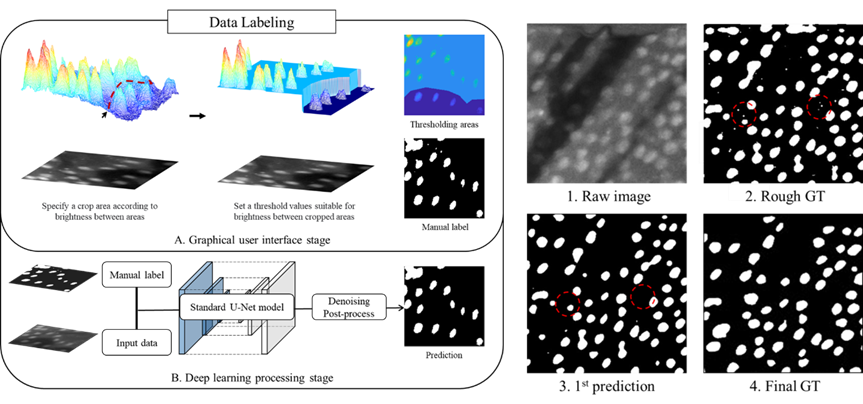


**Supplementary Figure 1.** Overview of the data labeling process. (A) represents the graphical user interface stage, while (B) denotes the deep learning processing stage. The outcomes of each stage are displayed on the right.
